# Acceptability and feasibility of conducting a pilot trial in Irish primary care: lessons from the IDEAs study

**DOI:** 10.12688/hrbopenres.13306.1

**Published:** 2021-07-14

**Authors:** Fiona Riordan, Katie Murphy, Colin Bradley, Patricia M. Kearney, Susan M. Smith, Sheena M. McHugh

**Affiliations:** 1 School of Public Health, University College Cork, Cork, Ireland; 2 Department of General Practice, University College Cork, Cork, Ireland; 3 Department of General Practice, Royal College of Surgeons in Ireland, Dublin, Ireland

**Keywords:** diabetes, primary care, pilot trial, feasibility study, diabetic retinopathy

## Abstract

Introduction

Understanding primary care practices’ ‘readiness’ to engage in trials and their experience is important to inform trial procedures and supports. Few studies report on the feasibility of study procedures though this is a central part of pilot trials. We explored the acceptability and feasibility of study procedures of a cluster randomised pilot trial of an intervention in primary care to improve uptake of Ireland’s national diabetic retinopathy programme.

Methods

As part of the embedded mixed-methods process evaluation, quantitative and qualitative data were gathered across four general practices participating in the intervention. Interviews were conducted with a purposive sample of staff. Research logs on time spent on intervention delivery, staff assignment, resources, problems/changes, and reasons for drop-outs, were maintained over the course of intervention rollout, and practice audit data were analysed. Quantitative outcomes included recruitment, retention, completion, and data quality and completeness. Qualitative data on perceptions and experience of the pilot trial procedures were analysed using the Framework Method.

Findings

Nine staff (3 GPs, 4 nurses, 2 administrators) were interviewed. An interest in the topic area or in research motivated practices to take part in the trial. Reimbursement meant they could ‘ *afford*’ to participate. Staff valued the researcher briefing at the start of the trial, to avoid ‘ *going in slightly blind’. *While staff varied in audit skills and confidence, and some found this aspect of data collection challenging, a ‘ *step-by-step’* audit manual and regular researcher contact, helped them stay on track and troubleshoot during data collection. Audit quality was acceptable overall, however there were some issues, incorrect assignment of patient status being most common.

Conclusion

The IDEAs trial procedures were acceptable and feasible for primary care staff, however, challenges with conducting the audit may reflect staff skills gaps and the need for greater guidance and support from researchers.

## Background

Strengthening primary care has been the focus of national ^1^ and international ^2, 3^ health service reform worldwide for decades. Quality improvement interventions and implementation strategies tend to be at the setting or system level, targeting patients via primary care health care professionals (HCPs) or HCPs directly. As a result, cluster randomised trials (cRCTs) in primary care practices have become more common ^4– 6^. There has also been more interest in pragmatic trials which focus on effectiveness in 'real world' settings like primary care ^7, 8^. Therefore it is increasingly important to understand what factors may affect trial conduct in this setting.

Practice capacity and available supports can influence the recruitment and retention of primary care practices for trials ^9– 11^ and research in general ^9, 12– 16^. Common factors reported to influence recruitment include clinician interest in the research topic ^9, 13, 15, 17– 20^, rapport between the practice and research team ^9, 17– 19^, and inability to commit time ^9, 13, 18, 20– 24^. Factors reported to influence retention include burden or ease of data collection ^9, 14, 20^, degree of clarity about the work involved ^18, 20^, compatibility of patient records systems ^18^, lack of time ^9^ or practice support staff ^18, 20^, and the quality of ongoing communication with ^9^, and support from ^18^, the research team. It is important to identify these issues early, and to assess and develop the ‘research readiness’ of primary care practices, which takes account of both capacity and willingness/motivation/attitudes ^25^. 

Efforts to build primary care research capacity and infrastructure have been bolstered by national investment in recent years, including the establishment of the Primary Care Clinical Trials Network Ireland in 2015 ^26, 27^. However, there are few reported examples ^21^ of trials in the Irish context which highlight issues related to primary care ‘readiness’. Few studies report on the feasibility of study procedures though this is an important part of pilot trials ^28^. As part of a cluster randomised pilot trial of an intervention in primary care, one of our aims was to examine whether the study procedures were acceptable and feasible for practice staff. We report our findings to highlight some of the challenges and lessons learned which are relevant to other researchers conducting trials in primary care.

## Methods

### Design

As part of a mixed methods process evaluation of the IDEAs (Improving Diabetes Eye-screening Attendance) intervention, quantitative and qualitative data were collected to assess the feasibility and acceptability of trial procedures.

A full description of the pilot trial methods is available elsewhere ^29^. In brief, IDEAs was a cluster randomised pilot trial with a wait-list control group, run over a 12-month period [July 2019-July 2020] in eight general practices; four intervention and four control ^29^. Expressions of interest (EOI) from practices were sought by distributing a recruitment flyer ^30^ through practice ^26, 31, 32^ and professional networks ^33^ and social media. Eligible practices had an electronic health record system and a practice nurse. Before random selection and allocation EOI practices were stratified according to size [number of fulltime practice nurses (large >1, small ≤1)], and deprivation ^29, 34^. 

The intervention comprised *both* professional-level components [a staff briefing, audit training (manuals and support), practice audit of patient screening status, HCP electronic prompt, and practice reimbursement of up to €1000 depending on total patients audited) and patient-level components [GP-endorsed reminders and an information leaflet delivered opportunistically face-to-face, and systematically by phone and letter] ^29^. Practices were provided with intervention materials, including a reminder letter template, an audit proforma in Excel or Word 2016 with the intention that the populated audit Excel file be returned to the research team. GP collaborators (SS, MM) and a Diabetes Nurse Facilitator (KM) piloted the audit process (e.g., 2–3 patients) using the audit Excel template and manual to check clarity, usability, and time required. 

### Data collection

To examine the feasibility of trial recruitment, retention and completion, characteristics of EOI practices, and data on study processes (time, staff assignment, resources, problems/changes, and reasons for drop-outs) were recorded by FR in research logs during the trial through regular ongoing contact with the intervention practices. 

To examine the feasibility of conducting the audit, we examined the quality and completeness of data returned by practices. Practices were given the target of auditing 100 patients with diabetes (type one or type two) aged ≥18 years, auditing a random sample if they had ≥100 patients with diabetes. They re-audited these patients at six months. We reported deviations from, or adaptations to, the data collection format, and omissions or errors.

To explore perceptions of the acceptability and feasibility of the main trial procedures we conducted semi-structured interviews at six months with a purposive sample of staff (a nurse and/or GP, and/or administrator per practice) from intervention practices who self-identified as being involved in intervention delivery ^35^.

### Data analysis

Quantitative data were managed and analysed using Microsoft Excel 2016 and Stata V14 software. Interviews were digitally-recorded and professionally transcribed verbatim. Transcripts were entered into NVivo qualitative analysis software to facilitate data management, coding, and retrieval. Interviews were coded using the Framework Method ^36^. *A priori* codes were largely descriptive (i.e., feasibility of intervention components), whereas codes related to acceptability were generated inductively from the data, through open (unrestricted) coding. Information in research logs and audit data quality were considered alongside staff perceptions of study procedures.

Ethical approval for this study was obtained from the Irish College of General Practitioners (ICGP) in April 2019.

## Results

### Practice recruitment and retention

In total, 60 eligible practices expressed an interest in the pilot trial, most of which were group practices located in urban areas ( Table 1). Eight practices were randomly selected from the 39 who responded during the 1-month recruitment period (March/April 2019). One practice which dropped out in the first two weeks was replaced. Upon starting the audit this practice found they would require significantly more time than could be allocated, partly as many patient records had relevant data in older handwritten charts. Subsequently, all eight practices were retained, completed the trial, and returned audit data.

**Table 1.** Profile of practices which expressed an interest in participating in the trial.

**N Practices**	60
Practice location *	
Rural	40%
Urban	60%
**Deprived area**	57%
**Practice staff**	
Single-handed GP	13%
Two GPs	30%
More than 2	55%

*assigned as per census definition of rural being a town with population <1500 and urban ≥1500

### Feasibility of the audit

Audit quality across the eight practices was acceptable overall, however there were some issues, incorrect assignment of patient status being most common ( Table 2).

**Table 2.** Audit data quality.

Type of issue	N variables and practices
Errors or omissions	
**Incorrect assignment of patient status** (e.g., recording people as ‘Recent non-attenders’ rather than ‘No record’ when they had no record on file)	3 variables, 4 practices
**Misinterpretation/recording different information** than intended (e.g., recording people as eligible to receive screening rather than eligible to receive the intervention)	6 variables, 3 practices
**Forgetting to record information** (e.g., recording yes/no whether patient was reached by phone)	3 variables, 2 practices
**Deviations or adaptations**	
Limited use of the drop-down menus, instead **populating columns/variables with alternative** **response categories** (e.g., recording screening attendance as yes/no rather than Attender/Non-attender/ Recent non-attender/No record)	2 variables, 1 practice
**Adding additional information** without definition or explanation (e.g., adding new columns to capture screening attendance dates)	1 variable, 1 practice

### Acceptability and feasibility of the study procedures

Data on research processes were collected across all four intervention practices. Ten staff who self-identified as being involved in intervention delivery were invited to take part in an interview, nine of whom participated: three GPs (Practice B,C,D), four practice nurses (A,B,C,D) and two administrators (B,D). 

### Motivation for taking part

Staff were willing to invest time in the study because of their own interest in diabetes specifically or research more generally, potential benefits for patients, and opportunity to fulfil mandatory professional competence requirements for an annual audit. Despite reimbursement, the study was not seen as a ‘ *paying procedure’* (GP#1-B), that is, something which would yield financial reward. The reimbursement, while a ‘ *positive thing’* (GP#3-D), factored into their decision only insofar as it meant they could ‘ *afford’* (GP#2-C) to engage in the study; *‘it wasn't loss of work from an appointments point of view’* (PN#1-A).

### Knowing what you are getting into

For staff, the study briefing was important to avoid ‘ *going in slightly blind’* (GP#1-B) to the trial, and ‘ *make sure we know what is going on’* (PN#1-A). Yet, at two practices, where the decision to take part was GP-initiated, the briefing was not sufficient. For example, one nurse (C), while ultimately determined the study was ‘ *doable*’, felt she would have benefited more from the briefing had she known in advance the practice was taking part. Another nurse felt it was only *after* the briefing, when they ‘ *started actually doing it [the study], that I realised what I could and couldn’t do*’ (PN#1-B).

Practice nurses were largely responsible for delivering the intervention and audit data collection, be it on their own or with some tasks completed by an administrator and GP. Staff felt it was important for everyone involved in the study to understand the nature of the investment required (i.e., skills, workload) *before* they decided to take part and to ensure these resources are in place. For example, understanding that they may need more advanced knowledge of the GP software, or assistance with aspects (e.g., preparing letters) where they lack the skills.

However, as mentioned, not all staff were involved in the decision to take part in the study, and all did not receive the explanatory information. The challenges of protecting time meant that just 21/52 (40%) of staff members attended the briefing with the researcher; in larger group practices, it was unfeasible for all staff to be available during the briefing time slot. While the intention when planning the study was to spend an hour in person, nurses were only able to commit 30 minutes to briefly go through the audit process and manual with FR.

‘ *Maybe you sent it to [GP] but maybe writing it out to the practice and saying “Okay we plan on doing this audit” and making sure that the person who might be doing the audit got that, before you came to visit.’* (PN#3-C)

### Support to stay on track

Staff highlighted the importance of ongoing support, represented by the study manual and researcher phone calls. The study manual was considered an informative ‘ *step-by-step’* (PN#1-A) reference, which was helpful for planning, to ‘ *figure out exactly what I was to do next’* (PN#1-A) and made staff feel the work was more manageable – *‘we couldn’t have done it without the manuals’* (Admin#1-B). For some, it bolstered confidence, circumventing the need for further local support (PN#4-D). Despite this, some aspects of the intervention were still missed. For example, one nurse missed an aspect of the intervention (e.g., the use of reminder letter template specified in the manual). She stressed it was important to fully read the study manual *‘to know exactly what is involved in it [study]*’ (PN#4-D)

**Figure 1.**
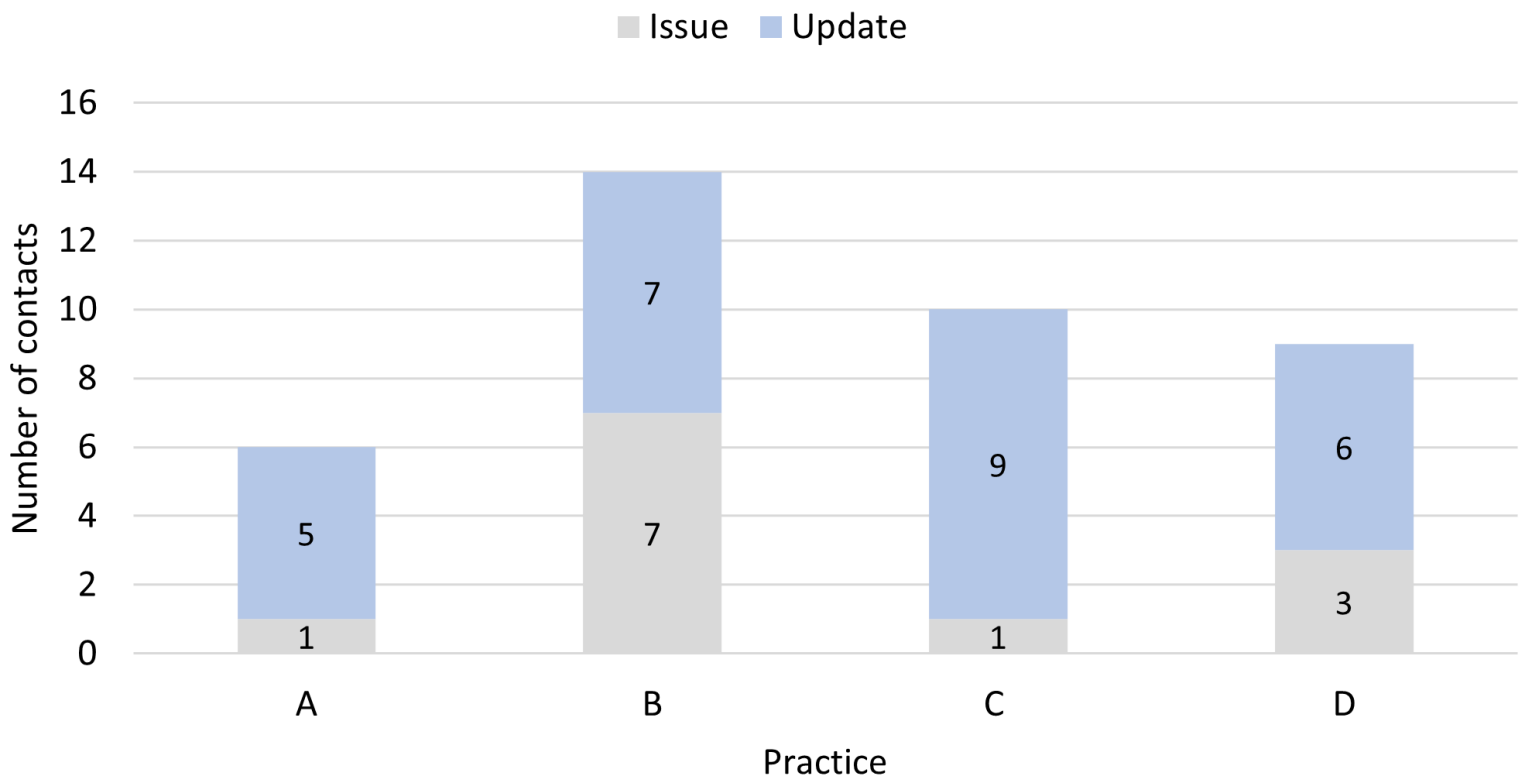
Number of contacts (email, text, or phone call) with the research team and purpose, whether to resolve an issue or provide an update. Note: most issues specifically related to the audit; 0/1 (A), 6/7 (B), 1/1 (C), 2/3 (D). Each contact took approximately five minutes except one contact at B which took an hour.

While support was valued across the board, nurses needed different *levels* of support to stay on track with delivering the intervention ( Figure 1); skills and confidence to conduct audit varied. The researcher liaised with practices during and beyond the end of the six-month intervention period to resolve issues with the Excel audit files (e.g., missing information, misinterpretation of variable headings).

In one practice (B) conducting the audit involved a lot of trial and error; they had a lot of difficulty with the ‘initial setup’, including running the searches on the GP software, and needed the most support. The reliability of coding in the GP software complicated implementation at this practice, making the initial searches challenging and necessitating ‘ *double checking’* (Admin#1-B). Staff recommended including a list at the start of the study manual summarising the steps and what was required:

‘ *I think maybe at the beginning of it [manual] should have the overall thing that you need done, rather than waiting. Because sometimes we didn’t have time to read through the whole thing again to see what’s the next stage, or what should we do next? If there was a really concise list of steps’* (Admin#1-B)

Such issues were reflected in the variation in self-reported time required to complete the audit [2.5 (C) to 23.5 hours (B)]. At the outset to avoid ‘ *a lot of time wasted’* staff felt it needed to be made clear that; 1) it was possible for things to go wrong – and the manual should provide ‘a *warning*’ (Admin#1-B) to that effect, and 2) extra support from the research team was available.

‘ *If it was already stated that “You may have problems with this; don’t worry, just ring this number straight away’* (Admin#1-B)

This support, the ongoing contact from a member of the team to populate the research log, served as a reminder to keep staff on track, and gave them confidence ‘ *you were there if I needed you’* (PN#1-D).

There were also delays with starting and finishing the audit due to the timing of the study; annual leave during Summer when the trial began and the busy flu vaccination period during intervention rollout (Oct./Nov.).

## Discussion

This study found trial procedures were acceptable and feasible to practice staff, highlighting the importance of dedicated time to brief staff on the trial, manuals, and regular contact with a readily accessible research team. Delays and challenges in this study related to, reach and clarity of the communication about study requirements, practice systems, audit skills, and study timing. We highlight two main implications.

Firstly, the findings suggest the importance of upfront discussion between researchers and practices about demands of taking part (i.e., protected time, skills and familiarity with GP software, disease coding, and Excel), who should be involved, cost implications, the timing of different stages of the trial including data collection, and the implications of any delays. In line with previous work, we found it is important to ensure all staff are aware of the study, informed and involved from the outset ^21^, in particular those who will be *delivering* the intervention whose workload will be affected. This early consultation could facilitate staff input on how the study can be operationalised successfully within the practice. There also needs to be clarity about the direct and indirect incentives (e.g. Continuing Professional Education credits) ^13, 19^ for the practice.

Secondly, the findings reflect the need for (a) local support from researchers throughout the study period, and (b) skills to conduct trials within primary care, particularly with respect to audit, our primary quantitative data collection method for this pilot trial. As with another trial in Irish primary care ^21^ we found continual contact between the research team and practice was important to troubleshoot and provide tailored support during and after the trial. Training was provided, but this was limited by practice nurse availability and it may be unfeasible to scale up the level of support for a full trial, which we estimate would require at least 50 practices. Alternative modes should be considered, for example, an online, on-demand briefing and training resources, along with FAQ, with an option to engage with a member of the research team virtually. Further consideration of the role of administrators in supporting trial data collection may also be worthwhile, developing their IT skills so they can lead or coordinate data extraction or act as a ‘go-to’ person for clinical staff. Greater usability testing of audit proforma with primary care professionals or reviewing a first draft of the data collection file from participating practices, may prevent or address errors, avoiding later troubleshooting. Ultimately, audit would be better facilitated through automated electronic processes, for example, whereby individual notifications about attendance come back to the practice automatically or collated information is provided by the screening programme, and data are easily and automatically extractable from patient records. Data may be more readily available through the new Chronic Disease Management programme ^37^, albeit on RetinaScreen referrals rather than attendance.

In conclusion, the IDEAs trial procedures were acceptable and feasible for primary care staff and patients, however, challenges with the audit data may reflect staff skills gaps and the need for greater or different guidance and support from the research team. While there is no conceptual model of primary care practice readiness to participate in trials, such a model could be valuable to guide researchers, for example, during early site meetings to pre-empt and proactively address issues or tailor support. If used at trial recruitment, such an assessment could contribute to our understanding of why practices participate or not, potentially identifying factors which consistently deter or preclude practices from taking part in trials. Consolidating learning from across trials in primary care would be a valuable starting point in developing such a model.

## Data availability

Some datasets generated and/or analysed during the current study (research logs, staff questionnaires, audit data, staff interviews) are not publicly available as the data pertains to the organisation of a very small number of general practices in Ireland. Limitations are based on the ethical approval received. Copies of study materials are publicly available on Zenodo, including:

Zenodo. IDEAs study information sheets and consent forms. DOI: http://doi.org/10.5281/zenodo.4337623 ^38^

This project contains the following underlying data:

- Information sheets and consent forms provided to practice staff and patients as part of the IDEAs pilot trial and process evaluation.

Zenodo. IDEAs study topic guides. DOI: http://doi.org/10.5281/zenodo.4337104 ^39^

This project contains the following underlying data:

- Topic guides used as part of the IDEAs study process evaluation.

Zenodo. IDEAs study coding framework. DOI: http://doi.org/10.5281/zenodo.4350281 ^40^

This project contains the following underlying data:

- Coding framework applied to staff and patient interviews conducted as part of the IDEAs process evaluation.

Zenodo. IDEAs study recruitment flyers. DOI: http://doi.org/10.5281/zenodo.4321202 ^41^

This project contains the following underlying data:

- Flyer used to recruit patients and members of the public to take part in a consensus process to develop the IDEAs intervention and/or to be part of a Patient and Public Involvement panel for the study.

Data are available under the terms of the Creative Commons Zero "No rights reserved" data waiver (CC BY 4.0 Public domain dedication).
