# Supplementary material for: Acceptability and feasibility of conducting a pilot trial in Irish primary care: lessons from the IDEAs study
Source: HRB Open Res. 2021 Jul 14;4:73. [Version 1] doi: 10.12688/hrbopenres.13306.1 (PMC8804907; doi:10.12688/hrbopenres.13306.1)
Supplement: Peer review history

# Peer review: Reviewer response for version 1

**DOI:** 10.21956/hrbopenres.14486.r31099

**Published:** 2022-01-31

**Authors:** Carlos Eduardo Leite Arieta, Daniel Borges

**Affiliations:** 1 Department of Ophthalmology and Otorhinolaryngology, Faculty of Medical Sciences, Universidade Estadual de Campinas, Campinas, Sao Paulo, Brazil; 2 Department of Ophthalmology and Otorhinolaryngology, Faculty of Medical Sciences,, Universidade Estadual de Campinas, Campinas, Sao Paulo, Brazil

The article explores the acceptability and feasibility of  the IDEAs trial procedures for primary care practices. By using quantitative and qualitative data, the authors show in detail the difficulties and feedback from the staff on various aspects of the trial. They give valuable information for other researchers on avoiding pitfalls and improving study trials' implementations.

 The paper is clearly written. The methods of data collection and analysis are adequate for the study design. Results and discussion answer the trial's primary objective. The authors declare that some datasets are not publicly available due to limitations based on the ethical approval received. Therefore, it is not possible to ensure full reproducibility of the study findings.

Is the work clearly and accurately presented and does it cite the current literature?

Yes

If applicable, is the statistical analysis and its interpretation appropriate?

Not applicable

Are all the source data underlying the results available to ensure full reproducibility?

Partly

Is the study design appropriate and is the work technically sound?

Yes

Are the conclusions drawn adequately supported by the results?

Yes

Are sufficient details of methods and analysis provided to allow replication by others?

Yes

Reviewer Expertise:

Ophthalmology

We confirm that we have read this submission and believe that we have an appropriate level of expertise to confirm that it is of an acceptable scientific standard.

# Peer review: Reviewer response for version 1

**DOI:** 10.21956/hrbopenres.14486.r30375

**Published:** 2021-11-08

**Authors:** Heather Barry

**Affiliations:** 1 Primary Care Research Group, School of Pharmacy, Medical Biology Centre, Queen's University Belfast, Belfast, UK

Thank you for asking me to review this interesting, clear, and well written manuscript, which reports the findings from mixed-methods process evaluation of a cluster randomised trial of a primary care intervention. I believe this will be of interest to researchers conducting similar trials of interventions in primary care and the findings are worth disseminating. I have made a number of minor comments below which the authors may wish to consider when revising the manuscript:

*Abstract*

1. The abstract lacks some key details and further clarification is needed in places:

- Introduction: What does the term ‘supports’ mean when used here?
- Methods: Please elaborate on ‘staff’ that comprised the purposive sample, even if only to clarify this meant both clinical and non-clinical staff.
- Methods: Quantitative outcomes included RATES OF recruitment, retention, completion, etc.
- Findings: What does ‘stay on track’ mean in this context?
- Findings: I also wasn’t clear what patient status meant here (in relation to patient status being incorrectly assigned).
- Conclusion: This is the first time that the study acronym has been used in the abstract – perhaps use and explain acronym earlier on in the abstract.
- Conclusion: What skills gaps?

*Background*

1. I think the introduction provides a concise background to the study, however just a little more detail would be helpful. In particular, whilst the authors have stated that there are few reported examples of trials in the Irish context which highlighting issues related to primary care readiness, it would be helpful to tease this out a bit more in terms of what is already known and where the gaps are exactly.
2. Line 5: The word ‘care’ after primary care can be removed here.
3. Line 12: What do you mean by ‘available supports’? Please clarify.

*Methods*

1. Design: Were GP practices recruited from all over Ireland or certain counties? Please clarify.
2. Design: Perhaps clarify the groupings of deprivation, similar to what has been done for size.
3. Design: GP-endorsed reminders about what?
4. Data collection: Can you clarify FR’s role in the research team similar to what you have done when you have mentioned other team members above (e.g. GP collaborators, nurse facilitator, etc.)
5. Data analysis: References are needed for software packages that were used, e.g. STATA and NVivo.
6. Please provide greater detail about what quantitative analyses were conducted.

*Results*

1. Practice recruitment and retention: Not all data on study processes (as mentioned in the methods section) are reported here.
2. Feasibility of the audit: I wasn’t totally clear on how feasibility was determined from the data that were described in the methods section.
3. Acceptability and feasibility of study procedures: The tense used in places here needs to be checked to ensure past tense is used, particularly under the ‘Knowing what you are getting into’ section.
4. Acceptability and feasibility of study procedures: Where the authors describe staff wanting a lit at the start of the study manual (under the ‘support to stay on track’ section) In wondered if this referred to a standard operating procedure?

*Discussion*

1. I think the authors make a valuable point about greater and clearer communication needed with those who will be delivering the intervention – whose responsibility is it to communicate with those involved in delivery?
2.  The comment about a conceptual model is an interesting one – again, are the authors able to tease this out a bit more in terms of what this should focus on and what further work is needed to work towards development of this?

Is the work clearly and accurately presented and does it cite the current literature?

Yes

If applicable, is the statistical analysis and its interpretation appropriate?

Not applicable

Are all the source data underlying the results available to ensure full reproducibility?

Yes

Is the study design appropriate and is the work technically sound?

Yes

Are the conclusions drawn adequately supported by the results?

Yes

Are sufficient details of methods and analysis provided to allow replication by others?

Yes

Reviewer Expertise:

Care of older people, prescribing and medicines optimisation, primary care, health services research

I confirm that I have read this submission and believe that I have an appropriate level of expertise to confirm that it is of an acceptable scientific standard.
